# Primary headaches in children and adolescents – experiences at a single headache center in Korea

**DOI:** 10.1186/s12883-018-1073-9

**Published:** 2018-05-21

**Authors:** Yun Jin Jeong, Yun Tae Lee, In Goo Lee, Ji Yoon Han

**Affiliations:** Department of Pediatrics, College of Medicine, Catholic University of Korea, Seoul, Republic of Korea

**Keywords:** Adolescents, Children, Headaches

## Abstract

**Background:**

Headache is a common complaint in children and adolescents. Recently, an increased prevalence of headache in children and adolescents has been reported.

**Methods:**

We retrospectively reviewed the medical records of children and adolescents attending the Headache Clinic of Daejeon St. Mary’s Hospital during the period from January 2005 through December 2016.

**Results:**

The study population consisted of 2466 children, aged between 3 and 18 years (mean age: 10.9). Our study showed an increase in the number of patients visiting the hospital with headaches during the past decade. Compared with 2005, the number of patients with headache increased three-fold in 2016. Interestingly, the proportion of boys, preschool children, and other primary headaches revealed a steady and statistically significant increase.

**Conclusion:**

Due to a steady increase in pediatric headaches, the earlier the problem is recognized and properly diagnosed and a treatment plan is established, the greater the likelihood of a better lifelong outcome. Studies are needed to estimate recent trend in prevalence and to identify the demographic and socioeconomic factors predicting the occurrence of headache.

## Background

Headache is a common problem in children prompting referral to pediatric neurology departments, and the incidence even higher during adolescence. The prevalence of headache ranges from 37 to 51% in seven-year-old children, gradually increasing to 57–82% by age 15. Before puberty, boys are affected more frequently than girls, but after puberty, headaches occur more frequently in girls [1, 2]. Recently, an increased prevalence of headache in children and adolescents has been reported [3]. Headache may result in significant disability, including missed school days, and extra-curricular activities, suboptimal participation in regular activities, and loss of productivity. Providers including pediatricians, parents, and caregivers need to improve children’s quality of life and minimize distress.

The purpose of this study is to provide detailed information about distribution and characteristics of headache at a single pediatric headache center in Korea over 12 years.

## Methods

We retrospectively reviewed the medical records of children and adolescents attending the Headache Clinic of Daejeon St. Mary’s Hospital from January 2005 through December 2016; the data were collected at the initial visits. The patient’s age ranged from 3 to 18 years. None of the included children suffered from diseases potentially associated with secondary headache such as brain tumors, paranasal sinus diseases, febrile illness, other systemic diseases, and vision problems.

During their initial and follow-up visits to the pediatric headache center, all patients and parents were interviewed using a detailed structured format according to the *International Classification of Headache Disorders, Third Edition, beta version* (*ICHD-3 beta*) criteria and were asked to complete a written questionnaire and to write a headache diary. The interview, the questionnaire, and the headache diary included questions related to demographics (age at onset, course, location, quality, frequency, duration of episodes, and associated symptoms), patient and family’ medical history, and history of head injury. The diagnosis and classification of headache was according to the *ICHD-3* or *ICHD-2* (in children younger than 7 years) criteria applied to children and adolescents. We divided headaches into three groups: migraine, tension type headaches (TTH), and other primary headaches. Other primary headaches included trigeminal autonomic cephalagias, cough headache, exercise headache, thunderclap headache, cold-stimulus headache, external pressure headache, stabbing headache, nummular headache, hypnic headache, and new daily persistent headache.

We performed all statistical analysis using SPSS software version 21 (SPSS, Chicago, IL, USA) and conducted comparisons using the Chi square and t-tests statistics. We used Pearson’s correlation coefficient to determine relevance. We considered probability values less than 0.05 as statistically significant. The study was approved by the Instituttional Review Board of the Catholic University of Korea.

## Results

The study population consisted of 2466 children, aged between 3 and 18 years (mean age: 10.9), and included 1492 (60%) females and 974 (40%) males. The mean age of headache onset was 8.5 years. Among the types of headache, migraine showed the earliest onset (7.5 years) and tension headache the latest (8.5 years), and for other primary headaches was 8 years, and the age of onset according to the type of headache showed statistical significance (*P* = 0.016). Males were significantly younger at onset (7.1 years) than females (7.9 years; *P* = 0.025). The mean duration of follow-up was 14.80 ± 13.19 months, with a range of 12 to 95 months.

**Fig. 1:**
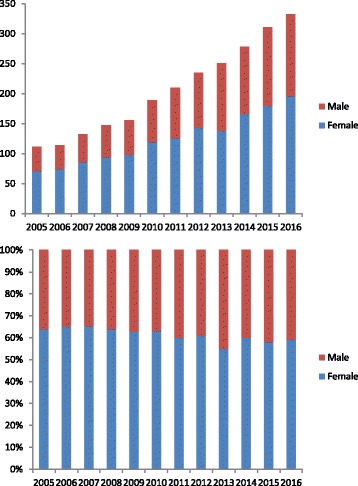
Number of patients with headache. **a** The increase over a decade, **b** The proportions by sex

The number of patients diagnosed with headache showed a steady increase during the past 14 years (Fig. 1a) from 111 patients in 2005 to 332 patients in 2016, a three-fold increase. The proportion of headaches in girls was significant higher than in boys (*P* < 0.009). The overall female-to-male ratio was 1.5:1, but the ratio in 2005 was 1.8:1 and it was 1.4:1 in 2016. The proportion of boys showed a steady and statistically significant increase (*r* = 0.985, *P* < 0.001; Fig. 1b).

**Fig. 2:**
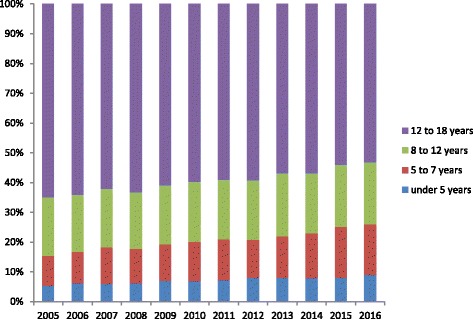
Patient proportions by age

The age distribution is shown in Fig. 2, with 530 (21%) children younger than 7 years, and 1936 (79%) of school age. The proportion of preschool children under the age of 7 increased from 15% at 2005 to 26% at 2016, which was s statistically significant increase over 12 years (*r* = 0.978, *P* < 0.001).

**Fig. 3:**
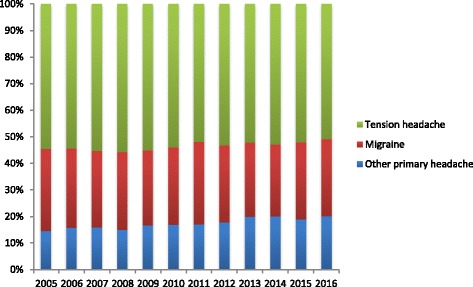
Patient proportions by headache type

During the study period, based on *ICHD-3* or *ICHD-2* criteria, 1056 patients (43%) were diagnosed with migraine, 863 (35%) had tension headache, and 547 (22%) had other primary headaches (Fig. 3). Other primary headaches included chronic daily headache (10%), cold-stimulus headache, primary stabbing headache (2%), primary exercising headache (1.5%), nummular headache (0.3%), primary cough headache (0.2%), and unclassified headache (8%). There was no statistically significant increase in tension-type headache or migraine during the study period, but other primary headaches revealed a statistically significant increase (*r* = 0.985, *P* < 0.001).

We compared the distribution of the different headache types in patients younger and older than 7 years of age (Table 1). In patients aged below 7, the most common types of headache were migraine (239, 45%), TTH (186, 35%), and other primary headaches (105, 20%) had other primary headaches. In patients aged above 7 years, 817 patients (42%) were diagnosed with migraine, 677 patients (35%) with tension headache, and 442 patients (23%) with other primary headache. The percentage of other primary headaches was higher in school children than in preschool children under 7 years old and the percentage of migraine was higher in preschool children. Other primary headaches and migraine were varied significantly in both groups (*P* = 0.048, *P* = 0.041, respectively). Eighty-one percent of parents of patients aged below the age of 7 years showed headaches and headache was detected in 65% of parents of headache patients aged 7 years and older. Positive parental history of headache in preschool children was statistically significant (*P* = 0.012). Aura symptoms were uncommon in migraine (15%, Table 2). The most common features of migraine were aggravated by phonophobia and photophobia. In general, other primary headaches were associated with higher frequency of symptoms than TTH.

**Table 1:** Headache comparisons between preschool age (younger than 7 years) and school age (older than 7 years)

Headache type	Preschool age (younger than 7 years) (%)	School age (older than 7 years) (%)	*P* value
Migraine	239 (45%)	817 (42%)	0.048
Tension headache	186 (35%)	677 (35%)	0.178
Other primary headaches	105 (20%)	442 (23%)	0.041
Parent’s headaches	429 (81%)	1258 (65%)	0.012

**Table 2:** Associated symptoms in different diagnoses

Associated symptoms	Migraine (%)	Tension type headache (%)	Other primary headaches (%)
Nausea/Vomiting	66	0	15
photophobia	75	5	25
phonophobia	72	4	35
Visual disturbance	15	2	7
Sensory symptoms	5	1	3
Speech difficulties	2	0	1

## Discussion

Headache influences children and adolescents of all ethnic and socioeconomic groups. It is common worldwide, occurring in up to 60% of children and adolescents aged between 2 and 18 years [4]. Headaches are a common complaint in childhood and adolescence and cause serious distress and disability in children and their families, resulting in reduced quality of life.

The most common type of recurrent headache in young children is migraine [5]. The frequency of tension type headache increases in late childhood, when TTH is the most common type of primary headache (20–25%), followed by migraine (8%) [6]. Chronic TTH affects about 1% of children close to adolescence [7]. However, other primary headaches are rare and reliable data are very limited. In epidemiology, the onset of primary headache is often observed in childhood or adolescence and the prevalence increases with age [8]. These progressions of headaches in both of the number of patients and the typical symptoms, increases the impact of headaches through the developmental period. After menarche girls are more likely to have headache than boys and even more so as they get older [9]. Last year, 17% of children in the United States had reported having frequent or severe headache [10]. According to a systemic review, 58% of children experience headache at least occasionally [4]. In a Korean study in 2009, the prevalence of headache among school children was 29.1%; by headache types, the prevalence was 8.7% for migraine, 13.7% for TTH, and 6.7% for other headaches [11].

In a Finnish study, the prevalence of migraine in seven-year-old children showed a three-fold increase over a period of 18 years (1.9% in 1974 to 5.7% in 1992), and the overall prevalence of headache increased nearly fourfold (14 to 52%) [3]. In a Swedish study published in 2003, the prevalence of tension type headache in children ages 7 to15 increased 2.3-fold from 9.8% in 1995 to 23% in 2003 [1]. And migraine prevalence rates increased considerably from 11 to 17%. In this population-based study, 1371 school children were aged 7–15 years, and the prevalence of headache increased with age, especially in girls. In an epidemiological study, recurrent primary headaches of childhood showed increasing prevalence and decreasing age of onset [12]. At preschool age, interrupting headache is uncommon, occurring in 4 to 20% of children in population studies [13]. At school entry, notably higher prevalence of headaches has been reported, ranging from 38 to 50% [3], which suggested the need to investigate the underlying stress factors and resolution of children’s problems. Furthermore, the precise diagnosis of headache in children achieved by improvement of ICHD criteria, so the incidence had trend to increasing.

A previous report showed ethnic and geographic diversity in headache prevalence [14]. This variation might be due to either miscellaneous cultural, genetic, or environmental circumstances or differences in methodology or diagnostic criteria. Methodological dissimilarities in symptom collections, history and ethnological, cultural, and geographical components probably accounted for differences in observation. Therefore, headache prevalence varies from country to country and from region to region in the same country.

Our study showed an increasing trend in the number of patients visiting the hospital with headaches over a decade, with nearly three-fold increase from 2005 through 2016. This rise in the number of patients with headaches shows importance of focusing on headache in the developmental course. We could not account for the exact cause of increased patients with headaches in this study, but it is well-known that academic career-oriented culture in Korea, especially among students preparing for the university entrance test is stressful contributing factor.

As in most studies, despite of a preponderance of headaches in females, the proportion of primary headaches in males, other primary headaches, and preschool children showed a significant increase over 12 years. Other studies found a significant increase in the prevalence of headache in children from 3 to 11 years, mostly during the shift from preschool to elementary school [10, 15]. This change may indicate a high stress burden and increased requirements for adaptive capabilities resulting in homoeostatic imbalances and in headache.

Children in early childhood cannot articulate their psychological and emotional status, so instead physical symptoms often appear as signs of stress. Many researches had shown that hardship during childhood correlates with persistent headache [16, 17]. Life adversities such as divorce, bullying, physical and sexual abuse are recognized as risk factors for chronic headache [18]. In preschool children, headache has been associated with low family socioeconomic status [19] and in adolescents, with divorced parents [20]. In a recent study of children younger than 6 years, the duration of headache attack was shorter than 30 min and associated symptoms, particularly phonophobia and photophobia, presented useful clinical factors [21]. Preschool boys experience recurrent headache more often than girls of this age [22]. Preschool age children may have difficulty explaining their symptoms, and in using terms for headache features. Therefore, parents and other care givers should remember this and take it seriously when they notice headache.

We found a significant association between parental headache and childhood headaches, especially in preschool patients. In one study, if a parent experienced headache, 72.3% of the children also showed headaches and in the absence of parental distress, the prevalence was only 27.7% of the children [9].

Headache is a multifactorial disorder compassing only genetic, medical and neuropsychological factors but also psychological and personal characteristics. The mechanisms that determine individual differences in headache sensation may be biological or psychosocial [6]. However, the nature and role of age-related factors in the discrimination of headaches and prognostic factors are still unknown. Headache can be a warning sign of stressful life and ill-being in children. Headache can be a warning sign of a stressful life, general ill-being, and social maladjustment of a child. Though literature is currently limited, factors suggested to correlate with headache include social change, stress, and physical activity level. Since children in low-income families are particularly more prone to headaches, early identification of these children is crucial for their long-term improvements.

Headaches in children and adolescents have devastating impact resulting in school absence, and decreased school performance, social withdrawal, and changes in family interaction. Personal stressors, changes in daily schedule, and sleep disturbances are recognized trigger factors of pediatric headaches [23]. In fact, the role of maladjusted stress response in headache has become of great interest in recent years [24], although the reasons for headache are not fully understand. According to Tarantino et al. children with severe migraine show propensity to retrain anger, whereas those with low migraine vent anger while suffering from separation anxiety [15]. Once headache was identified, patients should receive appropriate mental health care and medication. Successful management of headaches leads to improved quality of life and academic outcomes. Manage headache requires a multimodal treatment that combines bio-behavioral therapy with lifestyle modifications such as drinking sufficient water, exercising, keeping regular and healthy eating habits, and maintaining adequate and regular sleep. Recognizing pediatric and adolescent headaches and appropriate pharmacological and biobehavioral interventions can have long-lasting impact. Headache evaluation should include behavioural and mood co-morbidities such as depression and anxiety, because these conditions can negatively impact headache regulation. Headaches are mostly functional and result from psychological distress and emotional problems in children including attention deficit, depression, conduct disorder, eating disorders, and obsessive compulsive disorder. Data derived from additional studies are needed to show possible causal relationship and improve children’s quality of life.

We did not assess stressors using objective rating scales or evaluation of sleep or psychological co-morbidities. Multicenter studies may be of benefit to better understand reasons for increased pediatric headache patients. Our study is a retrospective, clinic-based study in a single pediatric headache center that might not represent the general pediatric population. Selection bias toward institutionalization was unavoidable. Future studies are needed to estimate the recent prevalence and to determine sociodemographic and socioeconomic factors to predict the occurrence of headache. In addition, additional investigation is needed to determine the biological mechanism and identify the factors that affect headaches in the pediatric period of life.

## Conclusion

This study showed a steady increase in the occurrence of headaches in pediatric patients. In particular, the proportions of boys, preschool children, and other primary headaches showed a steady and statistically significant increase. Children and adolescents with headaches should be diagnosed early and managed promptly to reduce headache-related affliction and enhance their quality of life. Furthermore, a future study is needed to understand biological mechanism and identify socioenvironmental factors that affect to headaches in pediatric period.

## Abbreviation

TTH: Tension type headache
